# Isolation of Kombucha Microorganisms for Application in Perilla Beverage Fermentation

**DOI:** 10.1002/fsn3.72254

**Published:** 2026-08-10

**Authors:** Nguyen Tri Yen Chi, Nhat Anh Duong, Tran Tuan Kiet Nguyen, Tran Quang Nho

**Affiliations:** ^1^ Department of Biotechnology, Faculty of Biochemistry and Food Technology Vinh Long University of Technology Education Long Chau Vinh Long Vietnam; ^2^ Department of Nutrition Vinh Long General Hospital Vinh Long Vinh Long Vietnam

**Keywords:** 16S/26S rRNA, bacteria, kombucha, perilla extract fermentation, yeasts

## Abstract

The increasing consumer demand for kombucha in Vietnam necessitates the development of standardized microbial starter cultures for functional beverage production. This study isolated and evaluated microbial consortia from five commercial kombucha samples using selective media: Yeast extract Peptone Glycerol Dextrose (YPGD) for acetic acid bacteria (AAB), MRS for lactic acid bacteria (LAB), and Yeast extract Peptone Dextrose Agar (YPDA) for yeasts. Based on morphological and biochemical characteristics, a total of 22 AAB strains, 6 LAB strains, and 9 yeast isolates were recovered. Sequence analysis revealed that selected strains shared high similarity with key species: *Komagataeibacter saccharivorans*, *Levilactobacillus brevis*, and *Wickerhamomyces anomalus*. Experimental evaluation with perilla extract revealed that a 15% sugar concentration combined with a yeast: AAB: LAB inoculation ratio of 2:2:1 performed favorably fermentation kinetics, with total flavonoid content (TFC) retention increased by 5%–10%, total anthocyanin content (TAC) elevated 300%–400% at day 3, and 2,2‐diphenyl‐1‐picrylhydrazyl (DPPH) antioxidant capacity maintained at 2.49 mg AAE/mL at Day 6 compared to other ratios. These results substantiate the potential of the isolated microbial strains as effective starter cultures for herbal beverage fermentation. Further studies on metabolic characteristics, sensory properties, and scale‐up are recommended to support industrial application.

## Graphical abstract

Five commercial kombucha samples from Vietnam were screened, yielding 37 isolates (22 AAB, 6 LAB, 9 yeasts). Three representative strains—*Komagataeibacter saccharivorans*, *Levilactobacillus brevis*, and *Wickerhamomyces anomalus*—were identified and combined into a defined starter consortium (yeast: AAB: LAB = 2:2:1). Applied to perilla extract fermentation (15% sugar), this consortium increased total flavonoid retention by 5%–10%, anthocyanin content by 300%–400% at Day 3, and maintained DPPH antioxidant capacity at 2.49 mg AAE/mL by day 6.

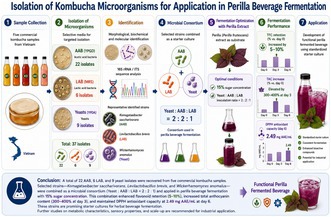

## 1 Introduction

The rising global demand for functional beverages has positioned kombucha as a prominent commercially available fermented tea, valued for its probiotic properties, antioxidant activity, and potential health benefits (Jayabalan et al. 2014; Villarreal‐Soto et al. 2018). In Vietnam, consumer interest in kombucha has surged dramatically, driven by growing health consciousness and shifting preferences toward natural, functional drinks. However, most domestic production still relies on undefined microbial consortia from previous batches or unstandardized commercial starters, resulting in inconsistent product quality, unpredictable fermentation profiles, and potential safety concerns (Coton et al. 2017). This variability underscores the critical need to develop standardized, region‐specific starter cultures to ensure reproducible fermentation kinetics and consistent functional properties for industrial‐scale production. Notably, recent research in Vietnam has highlighted the potential of lactic acid bacteria isolated from traditional fermented products to enhance kombucha's biological functions, yet comprehensive characterization of local commercial kombucha microbiota remains limited (Do et al. 2024; Tran‐Thi et al. 2026).

Kombucha fermentation is a complex symbiotic process primarily driven by the metabolic interactions between acetic acid bacteria (AAB), lactic acid bacteria (LAB), and yeasts (Costa Júnior et al. 2026; Khiabani et al. 2024). These microbial communities work synergistically to convert sucrose into organic acids, ethanol, and bioactive compounds that define the beverage's physicochemical characteristics and therapeutic potential. While several key species—including *Komagataeibacter*, *Lactobacillus*, and various yeast genera—have been identified in traditional kombucha systems, the microbial diversity and functional roles of strains present in Vietnamese commercial products remain largely unexplored. Recent studies employing selective media have successfully isolated viable AAB and yeast strains from commercial kombucha, demonstrating the feasibility of recovering viable microbial consortia for starter development (O'Sullivan and O'Sullivan 2024). Furthermore, species such as *Komagataeibacter saccharivorans*, *Levilactobacillus brevis*, and *Wickerhamomyces anomalus* have been identified as functionally important members of kombucha ecosystems, contributing to cellulose production and fermentation stability (Kim et al. 2025).

This study addresses these gaps by systematically isolating and characterizing microbial consortia from five commercial kombucha samples available in the Vietnamese market. Using selective media and molecular sequencing, we identified representative functional strains and evaluated their potential as defined starter cultures. Subsequent optimization experiments employed perilla extract as a model herbal substrate. We aimed to determine the ideal microbial inoculation ratio for enhanced fermentation performance, building upon recent work that highlights the importance of optimization for functional compound production (Jeong et al. 2024). The use of herbal extracts extends the traditional tea base and aligns with emerging trends in functional beverage innovation (Jakubczyk et al. 2020; Kawee‐Ai and Seesuriyachan 2019; Trivunović et al. 2025). The findings demonstrate the feasibility of developing standardized, effective starter cultures tailored for herbal beverage fermentation and provide a foundation for advancing industrial kombucha production in Vietnam and similar emerging markets, emphasizing the need for further evaluation of metabolic characteristics and sensory properties for industrial application (Andreson et al. 2022; Sanwal et al. 2023). By developing tailored starter cultures, this research provides a scientific foundation for standardizing herbal beverage fermentation, unlocking kombucha's potential beyond traditional tea, and addressing the critical gap in perilla fermentation research. While several studies have characterized kombucha microbiota globally, research in Southeast Asia remains limited. In Vietnam and neighboring countries, most production relies on back‐slopping methods with undefined consortia, leading to batch‐to‐batch variability (Do and Van 2025; Vo et al. 2024). Recent local studies have explored lactic acid bacteria from traditional fermented products, yet systematic isolation of defined starter cultures from commercial Vietnamese kombucha and their application to non‐tea herbal substrates such as *Perilla frutescens* have not been reported.

This study addresses these gaps by isolating and molecularly identifying dominant functional strains from Vietnamese commercial kombucha and optimizing a defined minimal starter consortium specifically for perilla beverage fermentation—a novel herbal substrate rich in thermolabile anthocyanins and phenolics. The work provides the first evidence of an optimized 2:2:1 (yeast:AAB:LAB) ratio tailored for herbal matrices, distinguishing it from conventional tea‐based systems.

## 2 Materials and Methods

### 2.1 Materials and Chemicals

Five branded Kombucha beverages were randomly purchased from local supermarkets in Ho Chi Minh city, Viet Nam. Chemicals and microbiological media included MRS broth (de Man, Rogosa and Sharpe), YPGD (Yeast Extract Peptone Glycerol Dextrose) broth, and YPDA (Yeast Extract Peptone Dextrose Agar) (Himedia, India); Gram staining kit, catalase and oxidase reagents (Nam Khoa Biotech, Vietnam); 2,2‐diphenyl‐1‐picrylhydrazyl (DPPH) (TCI, Japan); Folin–Ciocalteu reagent, Hydrochloric acid, Quercetin, and Ethanol (Merck, Germany); Gallic acid (Sigma‐Aldrich, USA); 3,5‐Dinitrosalicylic acid (Fisher Scientific, USA); and Sodium carbonate, Methanol, L(+)‐Ascorbic acid, Sodium nitrite, Sodium hydroxide, Sodium acetate trihydrate, Potassium sodium tartrate tetrahydrate, Potassium chloride, Aluminum chloride, and D(+)‐Glucose (Xilong Scientific, China).

### 2.2 Methods

#### 2.2.1 Isolation of Bacteria and Yeast Strains From Kombucha Samples

A 1 mL kombucha sample was separately inoculated into MRS broth (for LAB) and YPGD broth (for AAB), each incubated at 30°C for 24 h (Villarreal‐Soto et al. 2020). The enriched cultures were serially diluted to 10^−3^, and 0.1 mL was spread on MRS and YPGD agar supplemented with 0.5% CaCO_3_, followed by incubation at 30°C for 36 h (Rohaya et al. 2025). Distinct colonies with CaCO_3_ dissolution halos were selected and streaked for purification. The isolates were examined under a microscope, and identified via Gram staining and catalase tests to differentiate LAB (Gram‐positive, catalase‐negative) from AAB (Gram‐negative, catalase‐positive) (Axelsson 2004; Sengun and Karabiyikli 2011). For yeast isolation, a separate sample was plated on YPD agar with 50 mg/L chloramphenicol and incubated at 25°C for 48 h. Preliminary classification of the isolated yeast strains to the genus level was performed based on fundamental morphological and biochemical characteristics, including colony morphology, cell morphology, budding pattern, the ability to ferment sucrose and glucose, and urease activity (Kurtzman et al. 2011).

#### 2.2.2 Molecular Identification of Bacterial and Yeast Strains

Genomic DNA was extracted from pure cultures using the AccuRive Bacteria DNA Prep Kit (EX‐DNA04.1 A; Khoa Thuong Biotech, Vietnam) according to the manufacturer's instructions. For bacterial identification, the 16S rRNA gene was amplified using primers 27F (5′‐AGAGTTGATCMTTCTCAG‐3′) and 1492R (5′‐GGTTACCTTGTTAGGACTT‐3′) (Lane 1991). For yeast identification, the ITS region was amplified using primers ITS1 (5′‐TCCGTAGGTGAACCTGCGG‐3′) and ITS4 (5′‐TCCTCCGCTTATTGATATGC‐3′) (White et al. 1990). The PCR reaction mixture consisted of 1× PCR buffer, 2.5 mM MgCl_2_, 200 μM of each dNTP, 2 U of Taq DNA polymerase, 10 pmol each of primer, and bacterial genomic DNA as the template. Thermal cycling conditions were as follows: initial denaturation at 95°C for 5 min; 30 cycles of denaturation at 95°C for 1 min, annealing at 55°C for 1 min, and extension at 72°C for 2 min; followed by a final extension at 72°C for 10 min. PCR products were sequenced and compared against reference sequences in the NCBI GenBank database using the BLASTn algorithm.

#### 2.2.3 Determination of the Inoculation Ratio of Acetic Acid Bacteria, Lactic Acid Bacteria, and Yeasts for Herbal Beverage Fermentation

To determine the optimal microbial consortium composition, perilla extract was produced and pasteurized at 80°C for 10 min according to Duong and Nguyen (2025), then cooled to room temperature before sucrose was added at a concentration of 15% (w/v) (Chi et al. 2026). The uninoculated control (F1) consisted of pasteurized perilla extract with 15% sucrose but without added starter culture, serving as a negative control to assess background microbial activity and spontaneous changes. For inoculated treatments (F2–F5), each microbial strain (yeast, AAB, and LAB) was individually adjusted to a cell density of 10^5^ CFU/mL in sterile physiological saline (0.9% NaCl) prior to mixing. The three strains were then combined volumetrically at different yeast:AAB:LAB ratios (1:1:1, 2:1:2, 2:2:1, and 3:2:3, v/v), and the resulting mixed starter culture was inoculated at 5% (v/v) into the perilla fermentation medium. Fermentation was conducted in glass bottles sealed with breathable cloth covers to allow gas exchange while preventing contamination, providing semi‐aerobic conditions consistent with traditional kombucha fermentation. No SCOBY pellicle was added; instead, a defined liquid starter culture comprising three selected strains (Y2‐2, A2‐5, and L2‐1) was inoculated at 5% (v/v), corresponding to a final inoculum density of approximately 5 × 10^3^ CFU/mL for each strain in the fermentation medium. The initial pH of the pasteurized perilla extract with 15% sucrose, prior to inoculation, was 5.17 ± 0.11 (Table 4, F1, Day 0). Fermentation was carried out at ambient temperature (25°C–30°C) for 3–6 days, reflecting typical environmental conditions in Vinh Long Province, Vietnam, to simulate practical small‐scale production conditions and ensure complete fermentation and stable product quality (Villarreal‐Soto et al. 2020). Temperature was monitored daily using a calibrated thermometer throughout the fermentation period. Metabolic kinetics were monitored at 0, 3, and 6 days by analyzing total soluble solids (TSS) with a refractometer (ALLA, France), pH with an inoLab pH 7110 m (WTW, Germany), lactic acid (g/L) via the Therner method, acetic acid (g/L), and ethanol (%) was determined using the dichromate oxidation method described by (Sriariyanun et al. 2019). The total phenolic content (TPC) was measured using the colorimetric assay with the method of Folin–Ciocalteu assay (Singleton and Rossi Jr 1965). The reducing sugars was quantified using a colorimetric method by (Miller 1959). The DPPH assay was performed according to the method developed by (Brand‐Williams et al. 1995). Determination of total flavonoid content (TFC) by (Chi et al. 2025). Total anthocyanins content (TAC) was determined by the pH‐differential method (Giusti and Wrolstad 2001). The absorbance of the reaction mixture was measured with spectrophotometer (UV/Vis 200 nm–830 nm, Eppendorf BioSpectrometer Basic, Germany). Each treatment was performed in triplicate (technical replicates), with three bottles per treatment (*n* = 3 × 3).

### 2.3 Statistical Analysis

All statistical analyses were performed using the Statgraphics Program (Statgraphics Centurion XV, Statgraphics Technologies Inc., Old Tavern Rd., The Plains, VA 20198, USA) and expressed as values of mean ± standard deviation (SD). Differences between independent variables were analyzed by parametric analysis of variance (ANOVA), followed by a comparison of mean values using the Duncan test with a significance *p*‐value < 0.05.

## 3 Results and Discussion

### 3.1 Isolation of Lactic Acid Bacteria From Kombucha Samples

**FIGURE 1:**
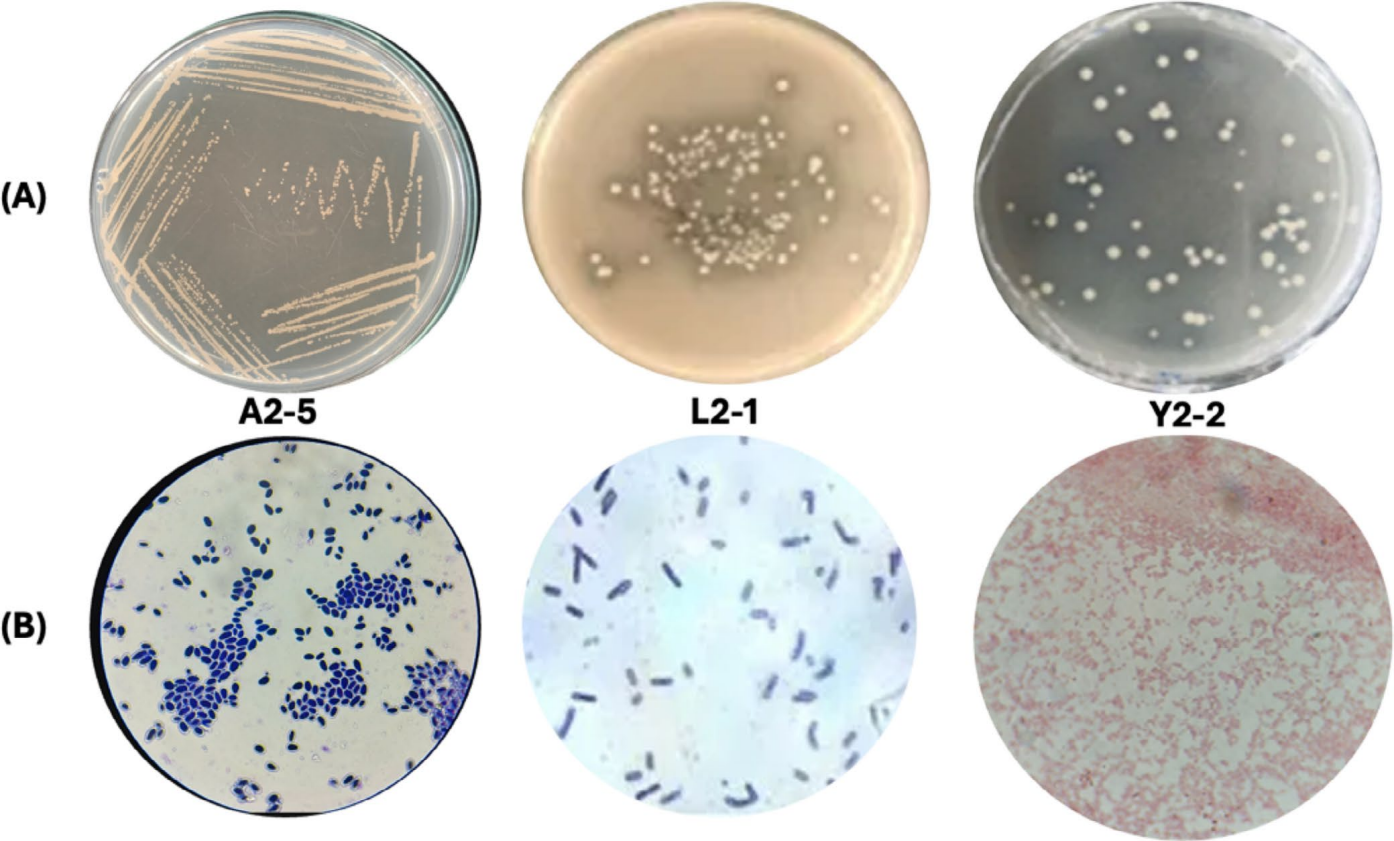
Colony morphology and microscopic characteristics of the representative kombucha isolates.

Screening of kombucha‐derived microorganisms yielded 37 isolates, comprising 28 bacteria and nine yeasts (Tables 1 and 2). Acetic acid bacteria (AAB) represented the most frequently recovered bacterial group under the selective isolation conditions applied in this study, with 22 isolates (78.6%) displaying Gram‐negative, catalase‐positive reactions and oval to short‐rod morphology, consistent with the characteristic AAB consortium in kombucha (Nam et al. 2025). In contrast, six isolates (21.4%) exhibited typical lactic acid bacteria (LAB) traits, including Gram‐positive, catalase‐negative, rod‐shaped cells, in agreement with previous reports on kombucha‐associated LAB (Li, Tso, et al. 2025). A minor subset (6.7%) showed atypical oxidase‐positive reactions. All nine yeast isolates presented uniform oval cells and smooth circular colonies, similar to kombucha yeasts described by (Wang et al. 2022). Representative colony and microscopic morphologies of the selected AAB, LAB, and yeast isolates are shown in Figure 1.

**TABLE 1:** Morphological, biological, and fermentation characteristics of 28 strains isolated from collected samples.

Isolate	Morphological		Biological		
	Colony morphology	Cell characteristics	Gram staining	Catalase test	Oxidase test
A1‐1	Round, convex, opaque white colony	Oval cells	−	+	−
A1‐2	Round, convex, opaque white colony with yellow center.	Oval cells	+	+	−
A1‐3	Round, convex, pale milky colony.	Oval cells	−	+	−
A1‐4	Round, convex, opaque yellow colony.	Oval cells	+	+	−
A1‐5	Round, convex, opaque white colony, small size.	Oval cells	−	+	−
A2‐1	Round, convex, opaque yellow colony.	Oval cells	−	+	−
A2‐2	Round, convex, pale yellow colony.	Oval cells	−	+	−
A2‐3	Round, convex, opaque white colony	Oval cells	+	+	−
A2‐4	Round, convex, opaque white colony, small size	Oval cells	−	+	−
A2‐5	Round, convex, pale yellow colony	Oval cells	−	+	−
A3‐1	Round, convex, pale yellow colony	Oval cells	+	+	−
A3‐2	Round, convex, opaque white colony with slightly yellow center	Oval cells, occurring in pairs or short chains.	−	−	−
A3‐3	Round, convex, opaque white colony	Oval cells	+	+	+
A3‐4	Round, convex, opaque yellow colony	Oval cells	+	+	+
A3‐5	Round, convex, opaque white colony with slightly yellow center	Oval cells	−	+	−
A4‐1	Round, convex, pale yellow colony	Oval cells	−	+	−
A4‐2	Round, convex, opaque white colony	Oval to round cells	+	+	−
A4‐3	Round, convex, opaque yellow colony	Oval cells	−	+	−
A5‐1	Round, convex, opaque white colony	Oval cells in pairs	+	+	−
A5‐2	Round, convex, opaque yellow colony	Oval cells	+	+	−
A5‐3	Round, convex, opaque white colony, small size	Oval cells	−	+	−
A5‐4	Round, convex, pale yellow colony	Oval cells	+	−	−
L2‐1	Round, raised, opaque white colony	Rod‐shaped	+	−	−
L2‐2	Round, raised, opaque white colony	Rod‐shaped	+	−	−
L4‐1	Round, raised, opaque white colony	Rod‐shaped	+	−	−
L4‐2	Round, raised, opaque white colony, large size	Spherical	−	+	+
L5‐1	Round, raised, opaque white colony	Rod‐shaped	+	−	−
L5‐2	Round, raised, opaque white colony, large size	Spherical	−	+	+

**TABLE 2:** Colony and cell characteristics of 09 strains isolated from collected samples.

Isolate	Colony description	Cell characteristics
Y1‐1	Circular, convex, cream‐colored	Oval
Y2‐1	Circular, smooth, cream‐colored	Oval
Y2‐2	Circular, smooth, pale yellow	Oval
Y3‐1	Circular, smooth, pale yellow	Oval
Y3‐2	Circular, convex, pale yellow	Oval
Y4‐1	Circular, smooth, cream‐colored	Oval
Y4‐2	Circular, smooth, cream‐colored	Oval
Y5‐1	Circular, smooth, cream‐colored	Oval
Y5‐2	Circular, smooth, pale yellow	Oval

Based on phenotypic traits and functional performance in preliminary screening—specifically, isolates displaying the Gram staining, catalase, and oxidase reaction profile, together with the colony and cell morphology, consistent with typical AAB (Gram‐negative, catalase‐positive, oxidase‐negative) or LAB (Gram‐positive, catalase‐negative) phenotypes as summarized in Table 1 were retained, whereas isolates showing atypical or inconsistent reactions (e.g., opposing Gram reaction, oxidase‐positive, or ambiguous catalase results) were excluded from further testing—, eleven AAB isolates (A1‐1, A1‐3, A1‐5, A2‐1, A2‐2, A2‐4, A2‐5, A3‐5, A4‐1, A4‐3, A5‐3), four LAB (L2‐1; L2‐2; L4‐1; L5‐1) isolates with consistent lactate‐producing profiles were prioritized for lactic acid contribution, and nine yeast (Y1‐1; Y2‐1; Y2‐2; Y3‐1; Y3‐2; Y4‐1; Y4‐2; Y5‐1; Y5‐2) isolates exhibiting typical ethanol producing morphology were chosen to represent the fermentative component. These 24 selected isolates will be subjected to acid production and ethanol content testing to validate their suitability for constructing defined starter cultures.

### 3.2 Acid and Ethanol Production Kinetics of Selected Isolates

**FIGURE 2:**
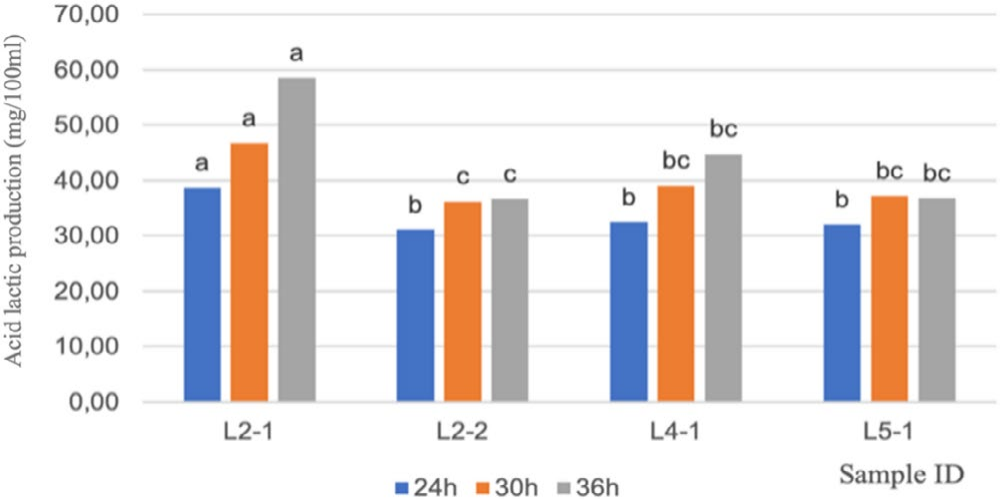
Lactic acid production by LAB isolates after 24, 30, and 36 h of incubation. Data are presented as the mean ± SD of three independent biological replicates. Bars of the same color with different lowercase letters are significantly different according to Duncan test (*p* < 0.05).

**FIGURE 3:**
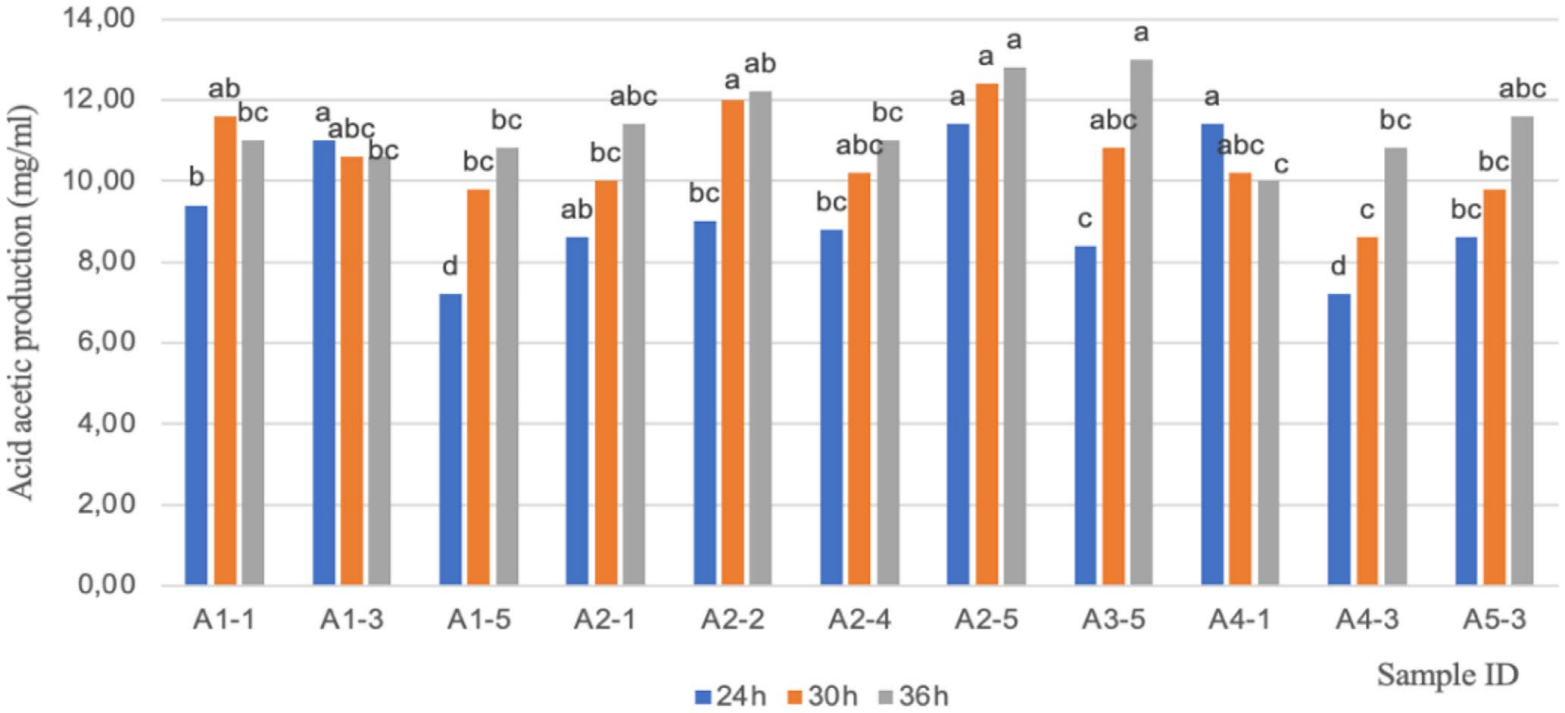
Acetic acid production by AAB isolates after 24, 30, and 36 h of incubation. Data are presented as the mean ± SD of three independent biological replicates. Bars of the same color with different lowercase letters are significantly different according to Duncan test (*p* < 0.05).

**FIGURE 4:**
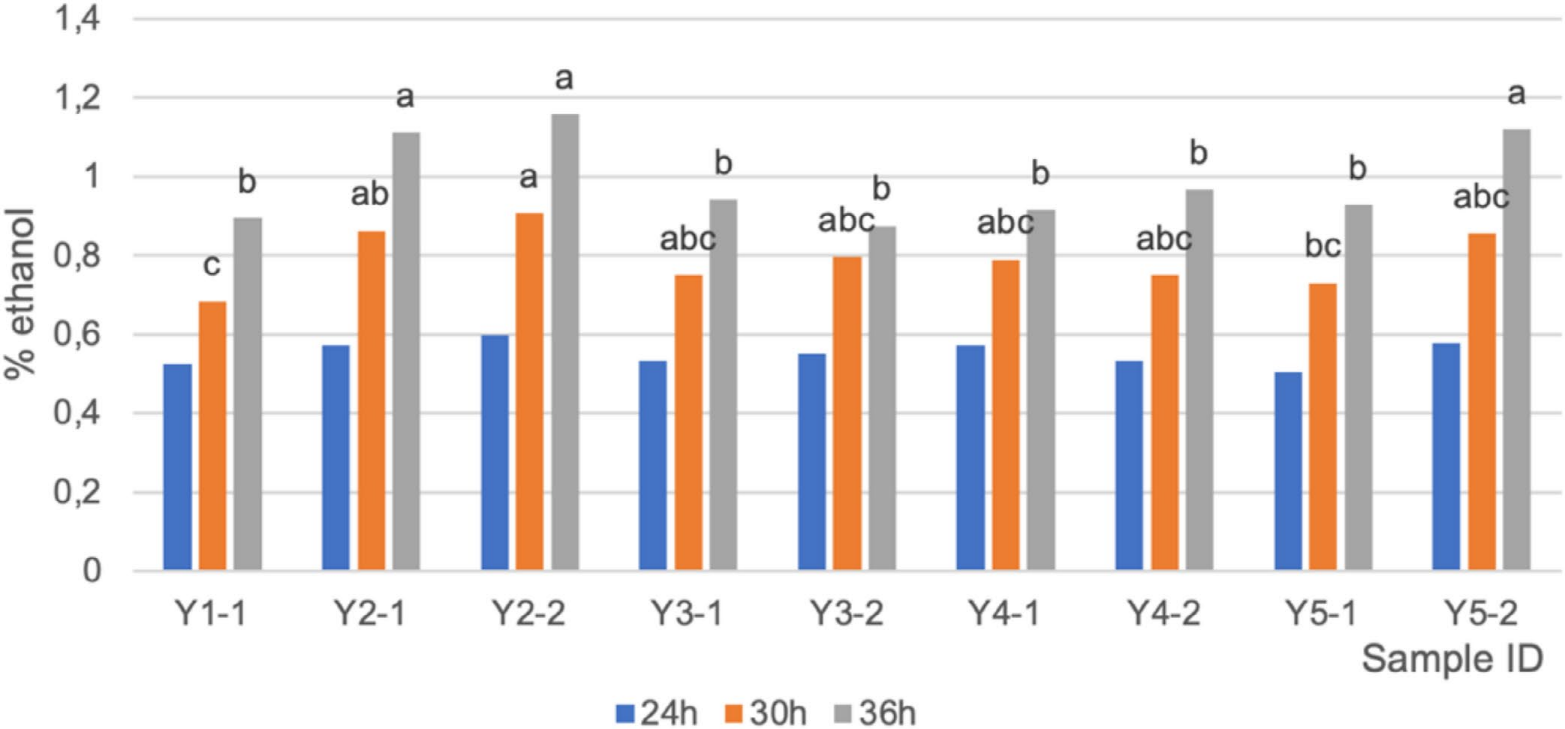
Ethanol production by yeast isolates after 24, 30, and 36 h of incubation. Data are presented as the mean ± SD of three independent biological replicates. Bars of the same color with different lowercase letters are significantly different according to Duncan test (*p* < 0.05).

The kinetics of acid and ethanol production by the selected isolates are shown in Figures 2, 3, 4. Lactic acid production (Figure 2) revealed that isolate L2‐1 (LAB) achieved the highest concentration of 58.5 mg/100 mL at 36 h, significantly surpassing other isolates (*p* < 0.05). Production followed a linear trend between 24 and 36 h, consistent with high‐performance kombucha lactobacilli (Laureys et al. 2020). Isolate A2‐5 (AAB) was selected for its acetic acid production, reaching 12.8 g/L at 36 h and maintaining stable synthesis after 30 h (Figure 3), allowing controlled acidification without compromising sensitive herbal substrates (Tefon Öztürk et al. 2023).

Ethanol production by yeast isolates (Figure 4) remained relatively low across all strains, with Y2‐1, Y2‐2, and Y5‐2 achieving 1.112%, 1.150%, and 1.119%, respectively, at 36 h. These values align with the ethanol‐limited environment of kombucha fermentation, where AAB rapidly oxidize ethanol to acetic acid (Bishop et al. 2022). Significant differences among yeast strains (*p* < 0.05) indicate strain‐dependent fermentation efficiency, and Y2‐2 was selected for its superior ethanol production.

Functional screening of 24 isolates over 36 h identified three optimal starter culture candidates (Figures 1, 2, 3): L2‐1 (LAB) achieved maximal lactic acid production, A2‐5 (AAB) yielded the highest acetic acid concentration, and Y2‐2 (yeast) demonstrated superior ethanol productivity while preserving kombucha's characteristic low‐ethanol phenotype (Wang et al. 2024). These three metabolically complementary isolates will undergo molecular identification and co‐fermentation trials to develop a defined minimal starter culture tailored for perilla beverage fermentation. A defined minimal starter culture refers to a standardized, reproducible consortium composed of selected, well‐characterized strains (in this case, one yeast, one AAB, and one LAB) at a specific inoculation ratio, as opposed to undefined back‐slopped SCOBY (Tran et al. 2022).

### 3.3 Molecular Identification of Bacteria and Yeast Isolates

Molecular identification of the three top‐performing representatives via 16S rRNA and ITS sequencing confirmed their affiliation with key kombucha taxa: isolate L2‐1 (LAB) showed 97.34% similarity to *Levilactobacillus brevis* (GenBank: PP446791.1), a species frequently reported in kombucha for its robust lactic acid synthesis and antimicrobial potential (Nguyen and Nguyen 2024); A2‐5 (AAB) exhibited 100% identity with *Komagataeibacter saccharivorans* (GenBank: CP036404.1), a well‐known acetifier known for efficient bacterial cellulose production and stable acidification kinetics (Kilmanoglu et al. 2024; Tran et al. 2020); and Y2‐2 (yeast) displayed 97.98% ITS sequence similarity to *Wickerhamomyces anomalus* (GenBank: MG183698.1), a yeast recognized for potentially contributing to its high ethanol yield (Qin et al. 2024). These molecular assignments corroborate the phenotypic screening results and align with frequently reported in kombucha microbiomes (Ben Saad et al. 2025), thereby validating the selection strategy.

The three confirmed isolates—representing lactic acid production (L2‐1), acetification capacity (A2‐5), and ethanol precursor synthesis (Y2‐2)—were subsequently used to determine the optimal inoculation ratio of acetic acid bacteria, lactic acid bacteria, and yeasts for herbal beverage fermentation, following experimental designs previously validated for kombucha‐derived consortia. The coexistence of yeasts, AAB, and LAB creates a synergistic fermentation system in which yeasts provide ethanol for AAB metabolism, while LAB contribute additional organic acids and bioactive compounds. These interactions collectively influence fermentation kinetics, microbial stability, and beverage quality (Koh et al. 2026; Li et al. 2026).

### 3.4 Effects of Inoculation Ratios

#### 3.4.1 Effects of Microbial Inoculation Ratio on Biologically Active

Temporal analysis of phenolic degradation revealed clear consortium‐dependent kinetics that underscore the substrate‐specific nature of herbal kombucha fermentation (Table 3). At baseline (Day 0), the uninoculated control (F1) established the initial phytochemical profile of perilla extract (TPC 31.32 mg GAE/mL; TFC 86.33 mg QE/mL; DPPH 2.44 mg AAE/mL), which then diverged markedly by Day 3 as fermentation progressed. F4 (2:2:1‐ yeast:AAB:LAB) preserved the highest TFC (78.26 mg QE/mL) and relatively elevated TPC (28.64 mg GAE/mL), an early stabilization attributable to AAB‐driven acidification that protects flavonoid–protein complexes (Içen et al. 2023; Pasquet et al. 2024) and consistent with reports linking a balanced yeast:AAB starter ratio to stable phenolic retention in kombucha fermentation (Liang et al. 2024). In contrast, balanced or LAB‐enriched consortia such as F2 (1:1:1) and F5 (3:2:3) exhibited accelerated flavonoid loss (TFC 74.23 and 63.53 mg QE/mL) and sharply reduced antioxidant activity (0.59 and 0.49 mg/mL), likely driven by synergistic β‐glucosidase and tannase activities previously documented in tea‐fermenting LAB systems (Mao et al. 2024) but demonstrably detrimental for herbal substrates. By Day 6, F4 solidified its superior performance, achieving the highest TFC (80.72 mg QE/mL), low residual reducing sugar (0.87 mg/mL; Table 4), and stable antioxidant capacity (DPPH 2.49 mg AAE/mL). This high antioxidant retention closely aligns with the progressive acidification observed in F4, where the pH dropped moderately from 4.08 on day 3 to 3.88 on day 6 (Table 4). Such controlled acidification helps protect flavonoid–protein complexes and stabilizes phenolic structures against oxidative degradation. Furthermore, this stability is consistent with literature suggesting AAB‐mediated conversion of glycosylated flavonoids into more bioavailable aglycones under mildly acidic conditions (Kumar et al. 2025). In contrast, formulations with more rapid or extreme pH drops (e.g., F2 and F5) exhibited accelerated flavonoid loss and reduced DPPH activity, likely due to acid‐induced hydrolysis and oxidative stress. Additionally, it is hypothesized that a reduced LAB fraction might minimize proteolytic degradation of flavonoid–protein complexes (Lugo‐Zarate et al. 2025) though specific microbial population dynamics were not quantified in this study. Meanwhile, F2 exhibited the greatest flavonoid reduction (TFC 58.44 mg QE/mL) and F5 the lowest DPPH activity (1.88 mg/mL), indicating destructive hydrolysis in LAB‐ and yeast‐rich systems. Although F1 displayed high DPPH activity (2.44 mg/mL), its concurrent TFC decline confirmed uncontrolled oxidation rather than true preservation. The DPPH assay measures the ability of antioxidants to scavenge stable free radicals, with higher values indicating stronger radical‐scavenging (antioxidant) capacity. The observed retention in F4 reflects protection of phenolic compounds through controlled acidification and reduced oxidative stress. Comparative kinetics thus position F4 (2:2:1) as the optimal starter ratio tested, with a metabolite profile broadly comparable to that reported for traditionally back‐slopped kombucha (Daval et al. 2024) and challenging the conventional 1:1:1 inoculum widely applied in tea‐based systems (Tran et al. 2022). These findings emphasize that herbal substrates require tailored starter ratios, favoring the yeast:AAB:LAB proportions tested here, to enhance phytochemical retention through optimized initial consortium ratios, rather than relying on formulations optimized for traditional tea substrates. The total anthocyanin content (TAC) exhibited substantial variation across formulations and fermentation stages. On day 3, formulations F3 (2:1:2) and F4 (2:2:1) reached the highest TAC levels (3.85–3.88 mg/L), representing an increase of approximately 300–400% compared with the initial TAC of F1 (0.99 mg/L). Such a marked elevation strongly suggests that higher inoculation levels—particularly the proportion of SCOBY derived from the original kombucha—facilitated enhanced anthocyanin release. This effect is likely driven by microbial enzymes such as cellulase, pectinase, and β‐glucosidase, which degrade plant cell wall structures and liberate bound anthocyanins during the early stages of fermentation (Dai et al. 2024; Samad et al. 2025). Similar observations have been reported in maqui juice and hibiscus kombucha, where microbial hydrolytic enzymes significantly increased anthocyanin extraction during the first 48–72 h of fermentation (Huang et al. 2022; Rocha‐Guzmán et al. 2023). By Day 6, however, TAC declined markedly across all formulations, showing a 30%–60% reduction relative to Day 3. This decrease is commonly observed in anthocyanin‐rich kombucha systems and can be attributed to oxidative degradation, polymerization, and structural transformation of anthocyanins under acidic conditions generated during active fermentation (Yang et al. 2023; Yuan et al. 2023). As fermentation progresses, decreasing pH and increasing microbial respiration accelerate anthocyanin breakdown, especially via hydration and cleavage of the flavylium cation (Li, Sun, et al. 2025; Ruta and Farcasanu 2019).

**TABLE 3:** Effect of starter ratio on the bioactivity of *perilla* kombucha.

Formulation code—day	TAC (mg/L)	TPC (mg GAE/mL)	TFC (mg QE/mL)	DPPH (mg AAE/mL)
F1 (0;0;0)–0 day	0.99 ± 0.08^e^	31.32 ± 0.92^a^	86.33 ± 0.30^a^	2.44 ± 0.17^a^
F1 (0;0;0)–3 day	2.12 ± 0.75^bc^	25.22 ± 0.44^de^	78.79 ± 0.18^c^	1.78 ± 0.47^ab^
F1 (0;0;0)–6 day	1.44 ± 0.07^de^	25.75 ± 0.33^cd^	76.16 ± 0.53^d^	2.46 ± 0.37^a^
F2 (1;1;1)–3 day	2.51 ± 0.43^b^	24.00 ± 0.72^fg^	74.23 ± 0.56^e^	2.50 ± 0.56^a^
F2 (1;1;1)–6 day	1.39 ± 0.13^de^	24.58 ± 0.32^ef^	58.44 ± 0.35^i^	2.32 ± 0.72^a^
F3 (2;1;2)–3 day	3.85 ± 0.14^a^	26.73 ± 0.53^c^	76.51 ± 0.20^d^	1.89 ± 0.31^ab^
F3 (2;1;2)–6 day	2.34 ± 0.68^bc^	25.97 ± 0.60^cd^	64.23 ± 0.48^f^	2.07 ± 0.22^ab^
F4 (2;2;1)–3 day	3.88 ± 0.28^a^	28.64 ± 0.58^b^	78.26 ± 0.37^c^	2.47 ± 0.41^a^
F4 (2;2;1)–6 day	1.82 ± 0.36^cd^	25.41 ± 0.51^de^	80.72 ± 0.30^b^	2.49 ± 0.55^a^
F5 (3;2;3)–3 day	1.30 ± 0.12^de^	26.59 ± 0.49^c^	63.53 ± 0.37^g^	1.48 ± 0.40^b^
F5 (3;2;3)–6 day	1.15 ± 0.15^de^	23.38 ± 0.36^g^	60.54 ± 0.44^h^	1.88 ± 0.17^ab^

*Note:* Data are presented as mean ± SD of triplicate analyses. Different letters in the same column indicate statistically significant differences (*p* < 0.05) by Duncan test.

**TABLE 4:** Effect of starter ratio on physicochemical properties of *Perilla* kombucha.

Formulation Code—day	TSS	pH	Reducing sugar (mg/mL)	Acid acetic (g/L)	Acid lactic (g/L)	Ethanol (%)
F1 (0;0;0)–0 day	16.00 ± 0.00^a^	5.17 ± 0.11^a^	1.34 ± 0.11^c^	0.30 ± 0.00^f^	0.45 ± 0.00^f^	0.00 ± 0.00^g^
F1 (0;0;0)–3 day	16.00 ± 0.00^a^	4.57 ± 0.03^b^	2.85 ± 0.23^b^	0.30 ± 0.01^f^	0.45 ± 0.01^f^	0.00 ± 0.00^g^
F1 (0;0;0)–6 day	16.00 ± 0.00^a^	4.38 ± 0.03^cd^	4.05 ± 0.36^a^	0.31 ± 0.01^f^	0.46 ± 0.01^f^	0.00 ± 0.00^g^
F2 (1;1;1)–3 day	15.67 ± 0.12^ab^	4.31 ± 0.04^bc^	0.59 ± 0.12^de^	7.00 ± 0.72^bc^	10.50 ± 0.26^c^	0.76 ± 0.03^f^
F2 (1;1;1)–6 day	15.00 ± 0.35^cd^	4.11 ± 0.09^d^	0.71 ± 0.28^cde^	7.80 ± 0.20^b^	11.70 ± 0.50^b^	1.88 ± 0.51^bc^
F3 (2;1;2)–3 day	15.13 ± 0.12^cd^	4.31 ± 0.04^c^	0.73 ± 0.39^cde^	6.80 ± 0.92^cd^	10.20 ± 0.44^c^	1.31 ± 0.34^de^
F3 (2;1;2)–6 day	14.73 ± 0.12^d^	4.13 ± 0.05^d^	0.71 ± 0.11^cde^	6.00 ± 0.60^d^	9.00 ± 0.90^d^	2.03 ± 0.29^abc^
F4 (2;2;1)–3 day	15.40 ± 0.20^bc^	4.08 ± 0.21^d^	1.16 ± 0.75^cd^	7.00 ± 0.40^bc^	10.50 ± 0.87^c^	1.06 ± 0.16^ef^
F4 (2;2;1)–6 day	15.13 ± 0.58^cd^	3.88 ± 0.22^e^	0.87 ± 0.26^cde^	9.70 ± 0.46^a^	14.55 ± 0.69^a^	2.38 ± 0.49^a^
F5 (3;2;3)–3 day	15.27 ± 0.12^bc^	4.29 ± 0.06^c^	0.49 ± 0.32^e^	4.80 ± 0.20^e^	7.20 ± 0.20^e^	1.60 ± 0.06^cd^
F5 (3;2;3)–6 day	15.13 ± 0.12^cd^	4.06 ± 0.13^d^	1.21 ± 0.30^cd^	7.80 ± 0.60^b^	11.70 ± 0.90^b^	2.17 ± 0.16^ab^

*Note:* Data are presented as mean ± SD of triplicate analyses. Different letters in the same column indicate statistically significant differences (*p* < 0.05) by Duncan test.

Among the tested formulations, F4 maintained TAC more effectively compared with F3 and F5, indicating that a balanced ratio of bacteria and yeasts contributes to anthocyanin stability. This may be linked to more moderate acidification dynamics and controlled oxidative stress within the fermentation matrix. Studies have shown that overly rapid acid production, often observed with high‐yeast inocula, accelerates anthocyanin loss due to enhanced redox cycling and enzymatic degradation (Enaru et al. 2021; Liu et al. 2024). Therefore, the performance of F4 suggests that the 2:2:1 mixing ratio offers an optimal microbial composition for maximizing anthocyanin extraction during early fermentation while minimizing degradation in later stages.

#### 3.4.2 Effects of Microbial Inoculation Ratio on Physicochemical Properties

The physicochemical properties of perilla kombucha were significantly influenced by the starter culture ratio and fermentation time (Table 4). The observed changes reflect the metabolic activities of symbiotic bacteria and yeast consortia (SCOBY), which drive the characteristic transformations during kombucha fermentation.

Total soluble solids (TSS) decreased progressively across all formulations containing SCOBY (F2‐F5) from Day 3 to 6 (*p* < 0.05), with a decrease of 3%–8% after 6 days of fermentation compared with the control (F1). The control formulation F1 (0;0;0) maintained constant TSS (16.00°Brix) throughout the fermentation period, confirming that microbial activity is responsible for TSS reduction. Formulation F4 (2;2:1) exhibited the most substantial TSS decline (15.40 → 15.13°Brix, −1.74%), while F2 (1;1;1) showed the highest reduction (−4.35%). This decline is characteristic of kombucha systems in which yeasts hydrolyze sucrose into glucose and fructose for biomass production and metabolite synthesis (Rodrigues et al. 2026), which are subsequently consumed by acetic‐acid bacteria (AAB) to produce organic acids (Huang 2024). Formulations F3–F5, which contained higher yeast inocula, exhibited the greatest reductions in TSS, consistent with accelerated sugar metabolism. The variation among formulations suggests differential sugar utilization efficiency influenced by the yeast: bacteria ratio, with balanced ratios (F4: 2;2;1) promoting more extensive carbohydrate metabolism.

All inoculated formulations demonstrated significant pH reduction (*p* < 0.05) during fermentation, dropping from initial values of 4.29–4.31 to 3.88–4.13 by day 6. The most pronounced acidification occurred in F4 (2:2:1), decreasing from 4.08 to 3.88 (−4.90%), indicative of optimal microbial acid production, highlighting the essential role of starter cultures in acidification. The pH reduction pattern aligns with lactic acid bacteria (LAB) activity, which dominates organic acid production during kombucha fermentation (Liao et al. 2024), where pH typically decreases due to progressive accumulation of organic acids produced by AAB and LAB (de Miranda et al. 2022). Acidification in kombucha is driven by the synergistic activity of both AAB and LAB, with AAB typically contributing significantly to acetic acid production (La Torre et al. 2025). The relatively lower pH in F4 suggests enhanced LAB metabolic activity, potentially due to the favorable 2:2:1 ratio that balances yeast‐driven ethanol production with bacterial acidogenesis. The uninoculated control (F1) showed a moderate pH decrease (from 5.17 to 4.38) and an increase in reducing sugars, suggesting limited spontaneous fermentation possibly due to residual endogenous microbiota in the perilla extract despite pasteurization (Brasileiro et al. 2026). This highlights the importance of using a defined starter culture to ensure controlled and reproducible fermentation (Gänzle et al. 2024).

Reducing sugar content likewise varied markedly among formulations: F1 (uninoculated control) accumulated the highest residual reducing sugar (4.05 mg/mL at day 6), consistent with its lack of microbial sugar consumption, whereas all inoculated formulations (F2–F5) showed markedly and comparably reduced residual reducing sugar by day 6 relative to F1 (0.71–1.21 mg/mL vs. 4.05 mg/mL), reflecting efficient substrate utilization by the microbial consortium regardless of the specific starter ratio.

Acetic acid content increased significantly with starter ratios, with acetic acid increased sharply in F4, F2, and F5, reaching 9.70 and 7.80 g/L, respectively, by Day 6—approximately 30%–220% higher than day 3 values, while F3 showed relatively lower production (6.00 g/L). This variation reflects the metabolic efficiency of Acetobacter species, which oxidize ethanol to acetic acid. Acetic acid was the key precursor for the generation of volatile compounds, especially esters (Wang et al. 2023) however, future volatile profiling is needed to confirm this in the perilla matrix. The superior performance of F4 correlates with its higher ethanol production (2.38%), providing ample substrate for acetic acid bacteria (AAB). These results are consistent with previous findings that optimal yeast:AAB ratios enhance acetic acid yield through efficient ethanol‐acetic acid metabolic coupling (Chong et al. 2024). Lactic acid production followed a similar trend, with F4 demonstrating the highest concentration (14.55 g/L on day 6, +38.57% from Day 3). Formulations F2 and F5 reached 11.70 g/L, while F3 produced the least (9.00 g/L). The elevated lactic acid in F4 suggests robust LAB activity Lactobacillus species, thriving in the acidic microenvironment created by initial acetic acid production (Han et al. 2024). The observed yeast:AAB:LAB ratio (2:2:1 in F4) indicates a balanced heterofermentative metabolism. This formulation supports strong synergistic activity between AAB and LAB, which contributes to extended shelf life and enhanced microbial safety of the kombucha (Al‐Kharousi 2025). Previous studies have similarly shown that inoculum ratio affects acid metabolism and can modulate both fermentation kinetics (Mendoza et al. 2007; Wang et al. 2025).

Ethanol production varied significantly among formulations, with all inoculated samples showing substantial increases by day 6. F4 (2:2:1) produced the highest ethanol content (2.38%), followed closely by F5 (2.17%) and F3 (2.03%). F2 exhibited moderate production (1.88%), while the control remained ethanol‐free. The superior ethanol yield in F4 (124.53% increase from Day 3) reflects optimal yeast activity facilitated by the 2:2:1 ratio. The control (F1) showed no detectable ethanol throughout, confirming that endogenous microflora alone cannot initiate fermentation. This is consistent with earlier findings that yeast abundance governs ethanol production, which in turn fuels AAB‐mediated acid synthesis (Davydenko et al. 2020; Dmytruk et al. 2025). The moderate ethanol levels (< 2.5%) observed in the present study fall within acceptable ranges reported for kombucha beverages (Jang et al. 2021). Regarding ethanol content (maximum 2.38% in F4), this level is typical for traditional kombucha and generally considered non‐alcoholic in many jurisdictions (< 0.5%–3% depending on regulations). However, in countries with strict limits (e.g., < 0.5% in some Muslim‐majority markets), further optimization or post‐fermentation dealcoholization may be required for broader commercial distribution (Burton et al. 2026). Ethanol serves as an essential precursor for subsequent acetic acid production, explaining the strong correlation between Day 6 ethanol and acetic acid levels across formulations (Cao et al. 2025).

These trends are consistent with the metabolic model of kombucha in which yeast generate ethanol and sugars are converted into organic acids via bacterial oxidation (Chen et al. 2025; Li, Tso, et al. 2025). Overall, formulations with a higher ratio of acetic acid bacteria (F2, F4, F5) favored rapid acidogenesis, leading to lower pH and higher acetic acid levels, while yeast‐dominant formulations (F4) promoted stronger ethanol and lactic acid accumulation. This optimal ratio likely reflects the natural symbiotic balance in mature kombucha consortia, where yeast (2 parts) provide sufficient ethanol substrate, while balanced bacterial populations (2 AAB: 1 LAB) efficiently convert intermediates to preservative organic acids. The 2:2:1 (yeast:AAB:LAB) ratio chosen over balanced (1:1:1) or bacteria‐heavy (2:1:2, 3:2:3) combinations lies in the specific metabolic dependencies of herbal kombucha systems. First, a higher proportion of yeast is essential to hydrolyze sucrose and generate sufficient ethanol precursors in an herbal matrix. Second, an equal proportion of AAB ensures efficient oxidation of this ethanol into acetic acid, creating a controlled acidic environment that protects thermolabile polyphenols and anthocyanins. Finally, restricting LAB to a lower proportion prevents excessive lactic acid accumulation and rapid drop in pH, which was shown in F2 and F5 to accelerate flavonoid degradation. Thus, the 2:2:1 ratio provides an optimal metabolic balance between ethanol synthesis and controlled acidogenesis for perilla extract fermentation. The data highlight the critical influence of starter ratio on fermentation dynamics and suggest that optimizing the microbial consortium plays a key role in acidity and physicochemical stability of perilla‐based kombucha beverages (Chi et al. 2026).

Although kombucha fermentation is generally recognized as safe, potential risks include contamination by spoilage organisms and excessive ethanol or acid production. The use of defined starter cultures in this study reduces such risks by providing a controlled, reproducible inoculum. This study has several limitations as quantitative microbial counts (CFU/mL) during fermentation were not determined, limiting direct assessment of growth dynamics and relative contributions of each group. Additionally, sensory evaluation and volatile compound analysis were not performed, although these are critical for evaluating flavor development and consumer acceptability.

The fermentation performance of the three representative isolates identified in this study was generally consistent with previous reports on these species in kombucha fermentation, although some differences were observed. *Komagataeibacter saccharivorans* A2‐5 produced 12.8 g/L acetic acid after 36 h, which falls within the range reported for *Komagataeibacter*‐dominated kombucha fermentations (approximately 6.6–16.4 g/L, depending on the substrate and fermentation conditions). This finding is consistent with the recognized role of *Komagataeibacter* spp. as an efficient acetifier and its frequent detection as a major AAB species in commercial kombucha communities. *Levilactobacillus brevis* L2‐1 produced the highest lactic acid concentration (58.5 mg/100 mL) among the LAB isolates evaluated, supporting previous reports that *L. brevis* contributes to acidification and may enhance the functional properties of fermented beverages through carbohydrate metabolism and biotransformation of plant‐derived compounds. In contrast, *Wickerhamomyces anomalus* Y2‐2 produced only approximately 1.15% ethanol after 36 h, despite this species being capable of substantially higher ethanol production under monoculture fermentation. The relatively low residual ethanol observed in the present study is likely attributable to the simultaneous oxidation of ethanol by the co‐cultured acetic acid bacterium, a characteristic feature of kombucha fermentation that favors organic acid accumulation while maintaining a low ethanol content. Overall, these findings indicate that the selected isolates performed according to their expected physiological roles within the microbial consortium. The results also suggest that the use of a defined three‐strain starter culture can provide a controlled and reproducible fermentation process while maintaining the characteristic metabolic balance of kombucha fermentation.

## 4 Conclusions

This study isolated and characterized promising microbial strains from Vietnamese commercial kombucha and identified a 2:2:1 (yeast:AAB:LAB) inoculation ratio with 15% sucrose that showed favorable fermentation performance and bioactive retention in perilla extract. The results provide preliminary evidence supporting the development of defined starter cultures for herbal kombucha production. However, further research including sensory evaluation, volatile compound analysis, microbial dynamics monitoring, and scale‐up studies is required before industrial application. To support scale‐up and industrial application, further research must also address starter culture stability during long‐term storage, reproducibility across larger batch volumes, and contamination control strategies.

## Author Contributions

**Tran Tuan Kiet Nguyen:** methodology, software, data curation. **Nhat Anh Duong:** software, writing – original draft. **Tran Quang Nho:** data curation, methodology. **Nguyen Tri Yen Chi:** methodology, investigation, writing – original draft, writing – review and editing, data curation.

## Funding

The authors have nothing to report.

## Ethics Statement

The authors have nothing to report.

## Conflicts of Interest

The authors declare no conflicts of interest.

## Data Availability

The data that has been used is confidential.
